# Disease progression in cardiac morphology and function in heart failure: ATTR cardiac amyloidosis versus hypertensive left ventricular hypertrophy

**DOI:** 10.1007/s00380-022-02048-5

**Published:** 2022-03-11

**Authors:** M. Y. Henein, B. Pilebro, Per Lindqvist

**Affiliations:** 1 Departments of Cardiology and Public Health and Clinical Medicine, Umeå University, Umeå, Sweden; 2 Departments of Clinical Physiology and Surgical and Perioperative Sciences, Umeå University, Umeå, Sweden

**Keywords:** Cardiac amyloidosis, Echocardiography, DPD scintigraphy, Prognosis

## Abstract

**Background:**

Transthyretin cardiac amyloidosis (ATTR-CA) is today more frequently recognized but the rate of progression of cardiac dysfunction is not well established. The aim of this study is to investigate the nature of cardiac structure and function changes, over time, in a retrospective cohort of ATTR-CA patients.

**Methods:**

Fifty-one patients with ATTR-CA (mean age 78 ± 7 years, 30 females) were compared with 20 patients with heart failure but no amyloidosis (HFnCA) (mean age 76 ± 7 years, 5 females), all with septal thickness >  = 14 mm. All patients underwent DPD scintigraphy and an echocardiogram (Echo 2) which was compared with a previous echocardiographic examination (Echo 1), performed at least 3 years before.

**Results:**

Over the follow-up period, the interventricular septal thickness (IVST) and relative wall thickness (RWT) in ATTR-CA increased from 16 (4) to 18 (5) mm and from 0.51 (0.17) to 0.62 (0.21) respectively, *p* < 0.001 for both, by a mean increase of 0.4 mm/year and 0.03 mm/year, (*p* = 0.009 and *p* = 0.002 compared with HFnCA), respectively. RWT > 0.45 (AUC = 0.77) and RELAPS > 2.0 (AUC 0.86) both predicted positive DPD diagnosis for ATTR-CA.

**Conclusion:**

In ATTR-CA patients, the overtime-increase in RWT and IVST was worse than that seen in patients with heart failure but no cardiac amyloidosis. Also, RWT and relative apical sparing predicted diagnosis of ATTR-CA, thus could strengthen the use of follow-up echocardiographic findings as red flag for the diagnosis of ATTR-CA.

## Introduction

Transthyretin (TTR) amyloidosis (ATTR) is the most common type of systemic amyloidosis and is classified into a hereditary type (ATTRv), and a wild-type transthyretin (ATTRwt) amyloidosis [1, 2]. One of the most common, and fatal manifestations of ATTR is cardiac amyloidosis (CA) [3]. The exact prevalence of ATTR-CA is unknown since it was previously considered a rare condition, but probably more common than presumed. [4–6]. Typical findings that raise the suspicion of ATTR-CA can be obtained from echocardiography, although the definitive diagnosis is always confirmed either by myocardial biopsy or non-invasively using nuclear scintigraphy [7, 8]. Typical echocardiographic findings suggestive of ATTR-CA include increased left ventricular (LV) wall thickness followed by development of restrictive ventricular filling patterns [9] and right heart involvement [10]. Furthermore, decreased global myocardial longitudinal strain (GLS) and increased relative apical sparing (RELAPS) have been reported as cardiac deformation signs associating ATTR-CA [11, 12]. Progression of cardiac amyloidosis is not adequately investigated. Recently, NAC (National Amyloidosis Centre) score including data from NT-proBNP and eGFR predicted clinical outcome in ATTR-CA [13]. Also, recent report by Itzhaki et al. showed that increased wall thickness and restrictive filling pattern develop over several years and can be seen prior to final diagnosis in patients with ATTR and AL amyloidosis [14].

The aims of this study were firstly, to investigate the ATTR-CA disease progression using echocardiography and compare it with that of heart failure patients with increased wall thickness but no CA (HFnCA), based on DPD scintigraphy. Secondly, to evaluate echocardiographic predictors for DPD scintigraphy verified ATTR-CA diagnosis.

## Materials and methods

### Patient population

This is a retrospective study in patients with heart failure and increased septal thickness. Inclusion criteria for further investigations in the patients were (1) septal thickness > 14 mm, (2) absence of types of CA other than ATTRwt and ATTRv, and (3) confirmed final diagnosis of ATTR amyloidosis, using 99mTc-3,3-diphosphono-1,2-propanodicarboxylic acid (DPD) scintigraphy examination. Part of this cohort findings was previously published [15]. We included 35 patients with ATTRv and 16 with ATTRwt from our local database, all diagnosed with ATTR-CA based on positive DPD scintigraphy (grade 2–3). In total, there were 51 ATTR-CA patients met the inclusion criteria for the study. For comparison of disease progression, we compared the ATTR-CA patients with 20 non-cardiac amyloidosis heart failure patients (HFnCA) with left ventricular hypertrophy, all due to primary hypertension, and none had positive DPD scan. Patients diagnosed with hypertrophic cardiomyopathy and aortic stenosis as causes for increased LV wall thickness were excluded from the HFnCA group. All patients and controls had clinical signs of heart failure and septal thickness > 14 mm on the Echo 2 examination.

Excluding AL amyloidosis in all patients was based on blood and urine analysis of serum free light chain (FLC) abnormalities (Freelite, Binding Site reagent, reference range 0.27–1.64) and the presence of monoclonal bands. Sequencing of the TTR-gene was also undertaken in all patients to diagnose ATTRv amyloidosis. Patients with Perugini score 1 were excluded from the study due to uncertain diagnosis. To evaluate the disease progression rate, we compared the two echocardiograms (Echo 1 and Echo 2) obtained using the same vendor. All patients had had an echocardiogram at the time of the DPD scan (Echo 2) and a previous echo (Echo 1) at least 3 years before.

### Echocardiographic examination

Echocardiographic examination was performed using a Vivid E9 system (GE Medical Systems, Horten, Norway) equipped with an adult 1.5–4.3 MHz phased array transducer. All Echo 2 studies were performed by the same investigator (PL) blinded to Echo 1 findings. Echo 1 studies were performed by various physiologists at the Clinical Physiology Department, Umea University. Standard views from the parasternal long axis-, short axis- and apical four-chamber views were used, following conventional protocols. Flow velocities were obtained using pulsed and continuous wave Doppler techniques as proposed by recent guidelines [16, 17]. All acquisitions were made from the left lateral position. Analysis was done blinded to patient’s category, ATTR-CA or HFnCA.

Relative ventricular wall thickness (RWT) was calculated according to the American society of echocardiography (ASE) recommendations (posterior wall thickness [PWT] × 2 / Left ventricular (LV) diastolic diameter [LVDd]). Trans-mitral blood flow velocities were obtained with the sample volume placed at the tips of the mitral valve leaflets with optimal angulation to LV inflow. From trans-mitral flow velocities, we measured early (E) diastolic and atrial contraction (A) flow velocities. Retrograde systolic trans-tricuspid flow was obtained from either parasternal right ventricular inflow or apical 4-chamber view, for measuring peak retrograde trans-tricuspid pressure drop using continuous wave Doppler, which reflects pulmonary artery systolic pressures. Left atrial volume was measured using the modified Simpson’s single plan calculation [4 chamber view] [18].Pulsed wave tissue Doppler velocities were also obtained to assess mean LV lateral and septal myocardial early diastolic velocities (e’) and E/e’ was calculated [18]. All Doppler recordings were made at a sweep speed of 50–100 mm/s with a superimposed ECG (lead II). Off-line analyses were made using commercially available software (General Electric, EchoPac version BT 13, 113.0, Waukesha, Wisconsin, US), and the means of three consecutive cardiac cycles were calculated.

### Assessment of LV deformation function

Anatomical landmarks were used, and care was taken for echocardiographic image acquisition to ensure adequate LV myocardium tracking, avoiding foreshortening of LV cavity when measuring global LV strain. Longitudinal myocardial deformation was assessed from the two-dimensional echocardiographic acquisitions using speckle tracking technology and was analyzed off-line. From the apical four chamber, two chamber and apical parasternal long axis views the endocardial border detection of the septal, apical and lateral LV walls was made manually, to analyze global LV strain (GLS) measurements. Strain recordings from three cardiac cycles were averaged, to assess GLS which were measured at end-systole with the reference point set at the onset of two consecutive QRS-complexes of the superimposed ECG. RELAPS was also calculated as average apical strain / (average basal strain + average mid cavity strain). Strain analyses were measured using a dedicated workstation (General Electric, EchoPac version BT 13, 113.0, Waukesha, Wisconsin, US). 2D strain assessment was done based on the recommendations of the European Association of Cardiovascular Imaging [19].

### DPD scintigraphy

All patients underwent DPD scintigraphy examination using an Infinia Hawkeye hybrid single-photon-emission computed-tomography gamma camera (SPECT-CT) (General Electric Medical Systems, Milwaukee, WI, USA,) with a low-energy high-resolution collimator. An intravenous injection of ~ 750 MBq DPD was given 3 h prior to the acquisition of whole-body planar image, followed by a non-contrast, low dose CT scan and a SPECT acquisition, 60 projections, iteratively reconstructed to a 128 × 128 matrix (OSEM, 3 iterations, 10 subsets) with scatter and CT-based attenuation correction. Reconstruction of SPECT images was performed on the Xeleris workstation (GE Healthcare, Waukesha, WI, USA). DPD scores were reported by two experienced clinicians using the Perugini’s grading system [20] with grade 0 being negative and grades 1 to 3 increasingly positive.

### Statistics

**Fig. 1:**
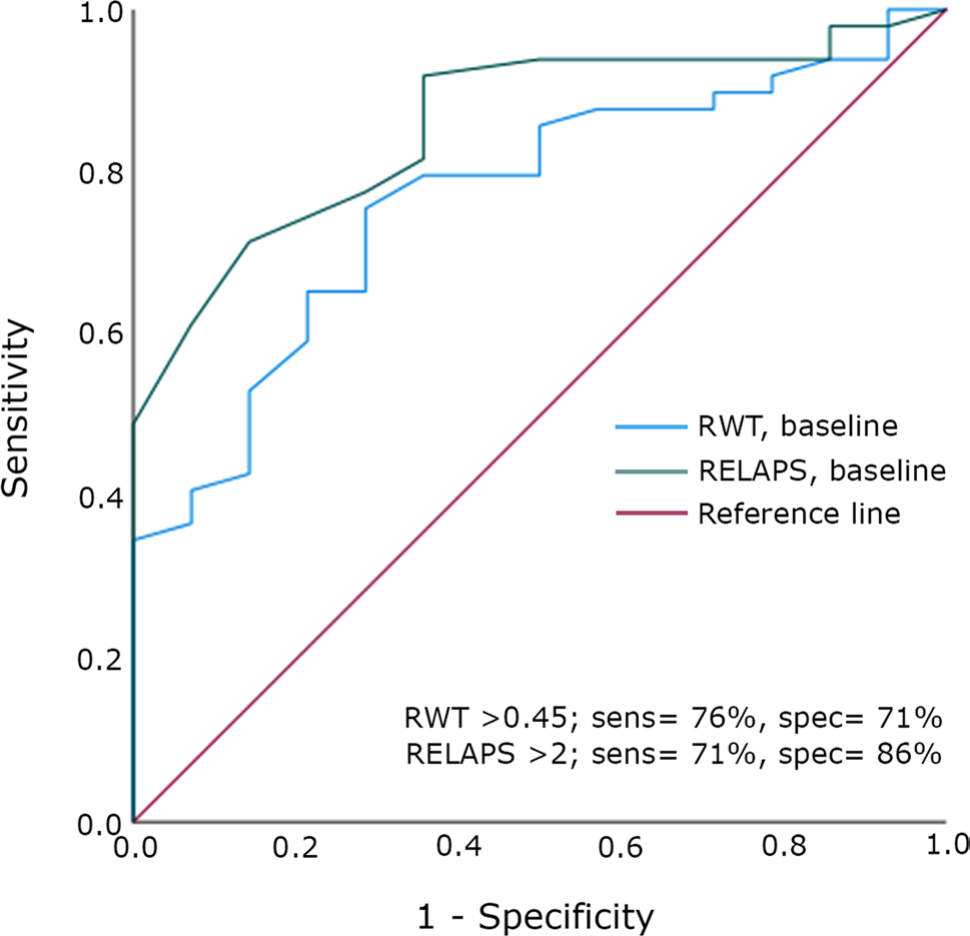
ROC curve testing relative wall thickness (RWT) and relative apical sparing (RELAPS) at baseline in predicting DPD positive ATTR-CA, RWT; AUC = 0.77, *p* = 0.002 and RELAPS; AUC = 0.86, *p* < 0.001

Statistical analysis was preformed using SPSS®, version 27 (IBM). Data are presented as mean, standard deviation (SD) if data were normally distributed, maximum and minimum values for continuous variables. Percentages were used to describe categorical variables. Categorical variables were compared using chi-square and McNemar tests, and continuous variables were compared using the Student’s t-tests for normally distributed data or Mann–Whitney *U* tests for non-normally distributed data. Wilcoxon paired test was used for comparing non-parametric data. Data were presented as median and interquartile range 25–75 for non-normally distributed data. Normality was assessed by Shapiro–Wilk’s test. ROC analysis was performed to evaluate the accuracy (area under the curve) and differentiating groups. P values were presented with a 0.05 level of statistical significance Fig. 1

.

### Ethics

The study complied with the declaration of Helsinki and the study protocol was approved by the Regional Ethics Committee of Umeå (reference numbers: 2016-435-31 M, 2018-418-32 M, 2018-137-32 M) and all subjects gave written informed consent to participate in the study.

## Results

Patient’s characteristics are presented in Table 1. Fifty-one patients with ATTR-CA (mean age 78 ± 7 years, 30 females) and 20 patients with heart failure (HFnCA) (mean age 76 ± 7, 5 females) were enrolled in the study. All patients had septal thickness >  = 14 mm. Follow-up time was longer in HFnCA than ATTR-CA patients (*p* < 0.001). All HFnCA patients had diagnosis of systemic hypertension as the cause for increased LV wall thickness. At the time of DPD, ATTR-CA patients had significantly higher NT-proBNP (*p* = 0.045), lower diastolic blood pressure (*p* = 0.046) and weight (*p* < 0.001), Table 1.

**Table 1:** Clinical data at the time for DPD verified diagnosis

	ATTR- CA (*n* = 51)	HFnCA (*n* = 20)	*P*-value
NT-proBNP log, ng/L	3.1 ± 0.6	2.8 ± 0.6	0.045
Troponin, ng/L	31.5 (35)	23 (19)	0.086
SBP, mmHg	130 ± 20	140 ± 17	0.061
DBP, mmHg	78 ± 10	84 ± 10	0.046
Height,cm	175 ± 8	175 ± 9	0.799
Weight, kg	78 ± 13	92 ± 21	< 0.001
Female sex (%)	30 (59%)	5 (29%)	0.017
Follow up time, years	6 ± 2	8 ± 2	< 0.001
NYHA, 2,3A,3B (%)	–	14/63/21	
Betablockers, *n*	20	19	
ACE/ARB, *n*	15	20	
MRA/Furosemid, *n*	12/12	6	
HFpEF, *n*	38	13	
HFmrEF, *n*	13	6	
HFrEF, *n*	3	1	

Abbreviations: *SBP* systolic blood pressure, *DBP* diastolic blood pressure, *ACE* angiotensin converting enzyme, *ARB* angiotensin receptor blocker, *MRA* mineral corticoid receptor antagonist, *HFpEF* heart failure with preserved ejection fraction, *mr* mid range, *r* = reduced

### Disease progression in ATTR-CA and HFnCA

There was no difference in septal thickness between the two patient groups at the time of Echo 1. At follow up, septal thickness increased significantly in ATTR-CA (*p* < 0.001) but not in HFnCA. LV posterior wall and relative wall thickness both increased only in ATTR-CA (*p* < 0.001. Left atrial volume increased in both groups but only significantly in ATTR-CA (*p* < 0.05). In the same group GLS decreased significantly (*p* < 0.001). 16% of ATTR-CA had AF at Echo 1 which increased significantly to 55% at the time of Echo 2. In comparison, only one patient with HFnCA had AF at Echo 1 and 12% had AF at Echo 2. The prevalence of AF at Echo 2 was significantly higher (*p* < 0.001) in ATTR-CA than HFnCA. RELAPS did not change over time in either of the two groups, Table 2

**Table 2:** Echocardiographic data at first and second echo

	ATTR-CA (*n* = 51)		HFnCA (*n* = 20)	
	First echo	Last echo	First echo	Last echo
Age, years	72 ± 7	78 ± 7*	68 ± 7	76 ± 7**
HR, bpm	74 (61)	70 (60)	66 (14)	69 (21)
IVS,mm	16 (4)	18 (5)***	16 (3)	16 (4)
LVDD, mm	45 ± 5	44 ± 5	51 ± 9	48 ± 7
PWT, mm	12 (3)	14 (3)***	10 (2)	11 (5)
LAV, ml	74 (54)	90 (115)*	60 (45)	114 (124)
RWT, mm	0.51 (0.17)	0.62 (0.21)***	0.45 (0.17)	0.3 8(0.44)
GLS, %	– 18.0 ± 4.8	– 13.2 ± 4.8*	– 13.1 ± 6.0	– 11.1 ± 2.8
RV-RA, mmHg	26 (8)	25 (15)	24 (3)	27 (31)
E velocity,cm/s	73 ± 25	62 ± 24	70 ± 20	62 ± 23
RELAPS	2.2 ± 0.6	2.0 ± 0.9	1.6 ± 0.5	1.4 ± 1.6
AF, %	8	27*	1	2

Abbreviations: *HR* heart rate, *IVS*  interventricular septum, *LVDD* left ventricular diastolic diameter, *PWT* posterior wall thickness in diastole, *LAV* left atrial volume, *RWT*  relative wall thickness, *GLS*  global longitudinal strain, *RV*  right ventricular. *RA*  right atrial, *E*  early diastolic, *RELAPS*  relative apical sparing, *AF*  atrial fibrillation, *p < 0.05 comparing first echo, **p < 0.001

### ATTRwt versus ATTRv

At the time of Echo 1, ATTRwt patients were older (*p* = 0.024), had thinner IVS (*p* = 0.03), larger LAV (*p* = 0.02) and lower LV GLS (*p* = 0.008) compared with ATTRv. At the time of Echo 2, RWT and RELAPS were lower (*p* = 0.019 and *p* < 0.001) in ATTRwt. The prevalence of AF was higher in ATTRwt at Echo 1 but lower at Echo 2. However, the prevalence of AF increased in both groups Table 3.

**Table 3:** Echocardiographic data at first and second echo in ATTRwt and ATTRv

	ATTRwt (*n* = 16)		ATTRv (*n* = 35)	
	First echo	Last echo	First echo	Last echo
Age, years	80 ± 5	84 ± 6*	69 ± 6	75 ± 6***
HR, bpm	77 ± 19	72 ± 12	76 ± 12	66 ± 13
IVS, mm	15 (5)	16 (3)*	16 (5)	20 (7)***
LVDD, mm	46 ± 6	45 ± 5*	45 ± 5	43 ± 5
PWT, mm	11.5 ± 1.6	13.0 ± 1.9*	11.9 ± 2.7	14.4 ± 3.2*
LAV, ml	80 (49)	82 (35)	50 (30)	79 (40)***
RWT, mm	0.54 (0.19)	0.57 (0.17) *	0.51 (0.20)	0.65 (0.20) ***
GLS, %	15 ± 5	12 ± 3.0	19 ± 4	14 ± 3
RELAPS	2.2 (0.8)	1.3 (0.7)***	2.3 (0.4)	2.2 (1.0)
AF, (%)	5	11*	2	15***
Female sex (n)	2		28	

Abbreviations: *HR* heart rate, *IVS*  interventricular septum, *LVDD*  left ventricular diastolic diameter, *PWT*  posterior wall thickness in diastole, *LAV*  left atrial volume, *RWT*  relative wall thickness, *GLS*  global longitudinal strain, *RV * right ventricular. *RA*  right atrial, *E*  early diastolic, *RELAPS*  relative apical sparing, *AF*  atrial fibrillation, *p < 0.05 comparing first echo; *p < 0.05 and ***p < 0.001 comparing first echo

### Disease progression rate in ATTR-CA and HFnCA

Since the time difference between Echo 1 and Echo 2 varied between patients, we investigated the rate of progression in echo measurements, such as RWT, RELAPS, GLS, RV-RA retrograde pressure drop and LA volume. We calculated the rate by dividing the difference in these measurements from Echo 1 to Echo 2 by the time difference between the two studies, in years. In ATTR-CA, septal thickness and RWT both increased by 0.39 mm and 0.023 mm/year, respectively which was significantly faster for both compared with HFnCA (*p* = 0.009 and 0.022). There was no difference between groups in the rate of progression of RELAPS, right ventricular-right atrial pressure drop or left atrial volume. The rate of fall of LV GLS was faster in ATTR-CA compared to HFnCA (*p* = 0.05).

### Disease progression rate in ATTRwt and ATTRv

The disease progression in ATTRwt and ATTRv was not different between groups, except for RWT that progressed faster in ATTvt (*p* = 0.012). Comparing ATTR-CA patients with or without hypertension showed no differences in disease progression rate, Table 4.

**Table 4:** Progression rate in different patient groups

	ATTR-CA (*n* = 51)	HFnCA (*n* = 20)	*P*-value
ΔIVS/year	0.39 (0.92)	0.25 (0.37)	0.009
ΔRWT/year	0.023 ± 0.035	0.003 ± 0.017	0.022
ΔRELAPS/year	0.065 (0.17)	0.030 (0.27)	1.000
ΔGLS/year	0.50 (1.3)	0.76 (1.2)	0.055
	ATTRwt (*n* = 16)	ATTRv (*n* = 35)	*P*-value
ΔIVS/year	0.57 ± 0.54	0.54 ± 0.51	0.844
ΔRWT/year	0.008 ± 0.02	0.029 ± 0.04	0.012
ΔRELAPS/year	0.06 ± 0.21	0.06 ± 0.18	0.878
ΔGLS/year	0.34 ± 1.4	0.88 ± 0.90	0.127
	ATTR-CA HT (*n* = 15)	ATTR-CA nHT (*n* = 34)	
ΔIVS/year	0.66 ± 0.62	0.52 ± 0.47	0.406
ΔRWT/year	0.021 ± 0.031	0.024 ± 0.038	0.831
ΔRELAPS/year	0.079 ± 0.20	0.061 ± 0.23	0.803
ΔGLS/year	0.60 ± 1.60	0.57 ± 0.78	0.363

Abbreviations: *IVS*  interventricular septum, *RWT*  relative wall thickness, *GLS*  global longitudinal strain, *RELAPS*  relative apical sparing, *HT*  hypertension

### Echocardiographic predictors of DPD verified ATTR-CA

Relative wall thickness and relative apical sparing predicted the diagnosis of ATTR-CA by DPD, RWT; AUC = 0.77, *p* = 0.002 and RELAPS; AUC = 0.86, *p* < 0.001, respectively. A RWT > 0.45 at baseline had a sensitivity of 76% and specificity of 71% in predicting ATTR-CA. Respective values for RELAPS > 2 were 71% sensitivity and 86% specificity in predicting ATTR-CA. Wall thickness did not predict DPD findings.

## Discussion

In two groups of heart failure patients with similar LV cavity morphology and wall thickness, our findings show significant differences in the rate of increased wall thickness changes over time, according to objective evidence for the presence of ATTR. Patients with ATTR-CA had faster rate of increased septal and relative wall thickness compared with HFnCA due to systemic hypertension. In the same group of patients with ATTR-CA, LV myocardial strain showed significant fall, more than that seen in HFnCA. Our findings also show a potential impact of the two types of ATTR-CA, with the wild type patients being older, frequently males, with larger left atrium and have faster rate of increased relative wall thickness compared to the non-amyloid. Hypertension in ATTR-CA did not seem to influence progression rate in cardiac function and morphology. Finally, RWT and RELAPS with a similar accuracy predicted the presence of ATTR-CA on the DPD scan.

It is well known that increased myocardial thickness is a common finding in patients with heart failure and preserved LVEF because of the increased prevalence of long-standing hypertension in these patients [21]. Recent studies have shown that cardiac amyloidosis is another common [22] cause of increased wall thickness in those patients, which when confirmed should be expected to influence treatment strategy [23]**.** We and others have reported a number of measures which assist in differentiating the two conditions of benign myocardial hypertrophy and infiltration as a causes for increased wall thickness [24]. Firstly, hypertension and obesity are more common findings in HFpEF than in ATTR-CA. Secondly, higher NT-proBNP is a common feature of ATTR-CA compared with HFnCA [25]. In this study, concentrically increased LV wall thickness (increased RWT) and relative apical sparing were more common in ATTR-CA compared with HFnCA. Interestingly, similar findings were found in our patients, up to 11 years before DPD verified diagnosis, with increased RWT and RELAPS predicting DPD diagnosis of ATTR-CA. A further detailed assessment of the pattern of increase of wall thickness showed that septal LV segments demonstrated faster progression rate of their thickness in ATTR-CA compared to HFnCA which was not paralleled in cardiac function measures (measured as GLS and RELAPS). This finding was independent of the type of ATTR-CA, ATTRwt or ATTRv. The same pattern of increase in septal thickness was significantly slower in HFnCA, a finding that might have significant implications in patient’s management.

The increase in wall thickness in our two heart failure groups of patients is fundamentally different. While the benign increase in wall thickness due to hypertension is caused by cellular hypertrophy and myocyte disarray, that in cardiac amyloidosis is mainly due to extra cellular amyloid deposition. The latter has been reported to be less pronounced in AL amyloidosis [26] but has more aggressive rate of progression which itself is known to carry prognostic value in AL amyloidosis patients [27]. The left atrium behaved differently in the two conditions, with its volume increasing more in ATTR-CA. The more pronounced increase in left atrial volume in ATTR-CA and especially in ATTRwt, is most likely related to the higher prevalence of atrial fibrillation in this group.

### Clinical implications

Our results show that both RWT and RELAPS could predict DPD-scintigraphy based diagnosis of ATTR-CA, a finding that has significant clinical relevance. It indicates the significant potential and importance of echocardiographic measurements in the diagnosis of ATTR-CA, with high accuracy, thus supporting its clinical role as red flag for ATTR cardiac amyloidosis. However, this potential relationship is limited since 12 of the ATTR-CA patients had septal thickness < 14 mm at baseline. Therefore, the proposed measures need to be re-tested in a larger cohort of patients. The unique application of RWT as an imaging marker for disease should be praised for being simple, universally available and does not need sophisticated expensive techniques. Its accuracy is also not dependent on rhythm patterns, as is the case with other investigations. In addition, a reduction in RWT and LV wall thickness and improved GLS has been found in ATTRv patients treated with RNA interference therapy [28] which strengthens the importance of assessing wall thickness and LV deformation pattern in ATTR-CA patients.

Limitations: The main limitation of this study is the relatively small sample volume; hence findings should be reproduced in a larger cohort before been considered for clinical guidance. The presence of atrial fibrillation could have influenced the results, especially those of LV function. We did not adjust for treatment, medical or liver transplantation, in either of the two patient groups which could have an influence on the results. However, we considered DPD verified evidence for ATTR cardiac amyloidosis an important differentiating evaluation of the disease progress.

## Conclusion

In ATTR-CA patients, the overtime increase in septal thickness was more profound than that seen in patients with heart failure but no cardiac amyloidosis. Also, relative wall thickness and relative apical sparing predicted diagnosis of ATTR-CA, thus could be considered as early echocardiographic markers for ATTR-CA. However, this findings need to be revalidated in a larger group of ATTR-CA.
